# Tumor response prediction in ^90^Y radioembolization with PET-based radiomics features and absorbed dose metrics

**DOI:** 10.1186/s40658-020-00340-9

**Published:** 2020-12-09

**Authors:** Lise Wei, Can Cui, Jiarui Xu, Ravi Kaza, Issam El Naqa, Yuni K. Dewaraja

**Affiliations:** 1 Applied Physics Program, University of Michigan, Ann Arbor, MI USA; 2 Department of Electrical Engineering, University of Michigan, Ann Arbor, MI USA; 3 Department of Radiology, University of Michigan, Ann Arbor, MI USA; 4 Department of Radiation Oncology, University of Michigan, Ann Arbor, MI USA; 5 Machine Learning Department, Moffitt Cancer Center, Tampa, FL USA

**Keywords:** ^90^Y, PET imaging, Liver cancer, Radiomics, Absorbed dose, Radioembolization

## Abstract

**Purpose:**

To evaluate whether lesion radiomics features and absorbed dose metrics extracted from post-therapy ^90^Y PET can be integrated to better predict outcomes in microsphere radioembolization of liver malignancies

**Methods:**

Given the noisy nature of ^90^Y PET, first, a liver phantom study with repeated acquisitions and varying reconstruction parameters was used to identify a subset of robust radiomics features for the patient analysis. In 36 radioembolization procedures, ^90^Y PET/CT was performed within a couple of hours to extract 46 radiomics features and estimate absorbed dose in 105 primary and metastatic liver lesions. Robust radiomics modeling was based on bootstrapped multivariate logistic regression with shrinkage regularization (LASSO) and Cox regression with LASSO. Nested cross-validation and bootstrap resampling were used for optimal parameter/feature selection and for guarding against overfitting risks. Spearman rank correlation was used to analyze feature associations. Area under the receiver-operating characteristics curve (AUC) was used for lesion response (at first follow-up) analysis while Kaplan-Meier plots and c-index were used to assess progression model performance. Models with absorbed dose only, radiomics only, and combined models were developed to predict lesion outcome.

**Results:**

The phantom study identified 15/46 reproducible and robust radiomics features that were subsequently used in the patient models. A lesion response model with zone percentage (ZP) and mean absorbed dose achieved an AUC of 0.729 (95% CI 0.702–0.758), and a progression model with zone size nonuniformity (ZSN) and absorbed dose achieved a c-index of 0.803 (95% CI 0.790–0.815) on nested cross-validation (CV). Although the combined models outperformed the radiomics only and absorbed dose only models, statistical significance was not achieved with the current limited data set to establish expected superiority.

**Conclusion:**

We have developed new lesion-level response and progression models using textural radiomics features, derived from ^90^Y PET combined with mean absorbed dose for predicting outcome in radioembolization. These encouraging, but limited results, will need further validation in independent and larger datasets prior to any clinical adoption.

**Supplementary Information:**

**Supplementary information** accompanies this paper at 10.1186/s40658-020-00340-9.

## Introduction

Delivering external radiation to multifocal/large liver tumors is a challenging task due to the damage of surrounding normal liver parenchyma. Hence, when disease burden is high, selective internal radiation delivery is preferred. Transarterial radioembolization (RE) with preferential delivery of glass or resin microspheres embedded with beta-emitting ^90^Y to hepatic tumors is an established treatment for unresectable hepatocellular carcinoma (uHCC) and liver metastases [1, 2]. Ability to predict lesion-level response immediately after therapy can facilitate adaptive therapies following RE by selecting lesion(s) predicted to be non-responding to the initial treatment for subsequent highly focal external stereotactic radiation.

Radiomics, a branch of quantitative image analysis, can capture heterogeneity characteristics of regions of interest (ROIs) by extracting relevant features from medical images (CT, MR, PET) and has been widely explored in the literature and shown to provide predictive capability of treatment response in different cancers [3–11]. Specifically, in patients undergoing transarterial ^90^Y radioembolization in uHCC, Blanc-Durand et al. showed that pre-treatment FDG-PET-derived radiomics features (strength for PFS, variance, strength, low intensity run short emphasis, and contrast for OS) for the whole liver are independent negative predictors for progression-free survival (PFS) and overall survival (OS) [12]. Gensure et al. found tumor contrast-enhanced CT-based texton and local binary pattern (LBP) features both achieve high accuracy in discriminating patient response to radioembolization (RE) with ^90^Y resin microspheres in terms of serologic response and survival status [13]. Recent studies by our group and others have reported on the association between post-therapy ^90^Y imaging-derived lesion absorbed dose and outcome (response, survival) in patients treated with ^90^Y radioembolization for primary and metastatic liver cancer [14–18]. However, to our knowledge, our current study is the first investigation to combine lesion radiomics features with absorbed dose metrics to predict outcome. Furthermore, our study relies on radiomics features from post-treatment ^90^Y PET imaging, unlike prior studies that used conventional FDG PET-derived features, which makes it unique in this respect. Compared with FDG-PET, ^90^Y PET is considerably more noisy due to the low true coincidence rate associated with a low yield positron in the presence of high random coincidence rates [19]. However, recent ^90^Y PET/CT studies have reported good quantitative accuracy and contrast-to-noise for dosimetry applications, using time-of-flight (TOF), longer acquisitions, optimized reconstruction parameters, and partial volume correction [18, 20]. Although ^90^Y can also be imaged by bremsstrahlung SPECT, the poor spatial resolution and challenges of correcting for bremsstrahlung scatter makes ^90^Y PET potentially better suited for radiomics analysis.

A major challenge of radiomics modeling especially with limited data is the robustness of the extracted features, as highlighted in recent review articles [21–23]. Variabilities can result from contouring, reconstruction algorithms, and filtering, even different scans with the same setting. Another challenge is the risk of overfitting when dealing with relatively small datasets. Therefore, in this study both issues are addressed by (1) conducting a phantom study to identify robust features, particularly to assess reconstruction and variability issues, and (2) applying a modified LASSO approach with bootstrap resampling for robust modeling. To mitigate analysis bias, nested cross-validation was used to train (feature selection, model construction) and test the outcome model (evaluation).

## Materials and methods

### Patient cohort

The study included patients with primary and secondary intrahepatic malignancies who had ^90^Y PET/CT imaging performed after ^90^Y radioembolization with glass microspheres (TheraSpheres) at the University of Michigan (UM) Medical Center as part of an ongoing dosimetry research study. Selection criteria for ^90^Y PET/CT imaging were well-defined lesions > 2 mL, ability to undergo imaging, follow-up at UM, and informed consent. The patient and lesion characteristics for the 36 lobar treatments (30 patients, 105 lesions, 6 patients had treatment to right and left lobes at different time points) are summarized in Supplemental Table 1. The treating physician followed standard guidelines to deliver 80–150 Gy to the treated liver with empirical adjustments within this range based on clinical factors. The ^90^Y PET/CT imaging was approved by the institutional review board, and all subjects signed an informed consent form.

### ^90^Y PET/CT imaging and dosimetry

Images were acquired on a Siemens Biograph mCT PET/CT within a couple of hours of the RE procedure (prior to discharge) with an acquisition time of ~ 30 min to cover the entire liver and partial lung. PET reconstruction parameters were selected based on phantom studies considering both activity recovery and noise: 1 iteration, 21 subsets of 3D OS-EM with time-of-flight and resolution recovery, and a 5-mm Gaussian post-filter [18]. The PET matrix size was 200 × 200 with a pixel size of 4.07 × 4.07 mm and a slice thickness of 3 mm. The CT was performed in low dose mode (120 kVp; 80 mAs) during free-breathing. The CT matrix size was 512 × 512 with a pixel size of 0.97 × 0.97 mm and a slice thickness of 2 mm.

PET images were transformed to CT-space, and the CT-derived density map was input to our DPM Monte Carlo code [18] to generate dose-rate maps that were converted into absorbed dose maps by accounting for ^90^Y physical decay. Mean absorbed doses to segmented lesions were reported following partial volume correction based on volume-dependent recovery coefficients, determined from a phantom study [18].

### Radiomics: lesion segmentation, PET data preprocessing, and feature extraction

Lesion segmentation was performed on diagnostic quality contrast-enhanced baseline CT or MRI by a radiologist specializing in hepatic malignancies (RK), which is considered a gold standard. Note that variability due to contouring can be a source of error but has been addressed in several previous studies [6, 24, 25]. The diagnostic scan was then rigidly registered to the CT of the ^90^Y PET/CT, and the contours were transformed with fine-tuning when misregistration was evident on MIM (MIM Software Inc, Cleveland, OH). In some cases, where the lesions were well visualized on the non-contrast low-dose CT of the PET/CT, they were directly defined on this CT in order to minimize misregistration effects. Up to 5 (largest) lesions > 2 mL were segmented per patient.

Lesion contours and ^90^Y PET images were input to an in-house-developed (Matlab, MathWorks Inc., Natick, MA) radiomics toolbox (benchmarked by image biomarker standardization—ISBI) that run as an extension on MIM. Our radiomics code is shared at https://github.com/mvallieres/radiomics. All subsequent analyses were performed in MATLAB. First, a root-squared transform was applied to the PET images to reduce quantum noise effects [26].

The full intensity range of the tumor region was quantized to a smaller number of gray levels (Ng) before computation of the features. The quantization algorithm used is Lloyd-Max algorithm, which attempts to minimize the mean-squared quantization error of the output. Ng was experimentally chosen as 32 [27]. The features were extracted from 3D ^90^Y PET images, which were interpolated to isotropic voxel size (0.97 mm). Forty-six features, including volume, one shape feature (sphericity), 4 global features, and 40 texture features from gray-level co-occurrence matrix (GLCM), gray-level run length matrix (GLRLM), gray-level size zone matrix (GLSZM), and neighborhood gray-tone difference matrix (NGTDM), were extracted. All the feature extraction followed the image biomarker standardization initiative (IBSI) guidance [28]. These features represent the spectrum of commonly used features, especially in PET imaging [6, 29, 30]. We further opted for extraction parameters following the ISBI guidelines due to the limited sample size; we did not explore further parameterization or less commonly used features. Supplemental Table 2 presents the list of radiomics features used in this study.

### Lesion-level study endpoints

Two endpoints applied at the lesion-level were considered: overall response (OR) classification at first follow-up and time to progression at lesion level. For OR assessment, diagnostic CT or MR at first follow-up was used by a radiologist (RK) to measure percentage reduction in lesion diameter relative to baseline according to RECIST criteria [31]. Lesions that met the RECIST criteria of partial or complete response were classified as responding (OR = 1), and others (stable or progressive disease) were classified as non-responding (OR = 0). For time to progression, clinical follow-up images and records were assessed by a radiologist (RK) and were defined as the time in months from the date of ^90^Y therapy to date of local progression. Lesions without evidence of progression were right censored at the last date of hepatic imaging. This included lesions that had not progressed at the time of death. Any lesion that had additional liver lesion-directed therapy after ^90^Y treatment was also right censored at the date of the additional treatment.

### Phantom study to assess radiomics feature repeatability and reproducibility

A ^90^Y PET/CT study with a liver/lung torso phantom consisting of a “warm” liver compartment and three “hot” lesion inserts (29-mL ellipsoid, 16-mL sphere, 8-mL sphere) with an insert-to-liver activity concentration ratio of 5:1 was performed. The total activity in the phantom was 1.9 GBq, and the activity concentrations in the inserts were 6.0–7.3 MBq/mL and liver minus inserts was 1.2 MBq/mL. To assess radiomics feature repeatability, 5 consecutive 30-min acquisitions under identical conditions were performed on the same PET/CT system as in the patient studies. To assess sensitivity to reconstruction parameters and filtering, each of the 5 scans were reconstructed with 1 and 2 OS-EM iterations (21 subsets) and with and without Gaussian post-filter. The activity concentrations, acquisition time, and parameters used in the phantom study were chosen to reflect conditions for imaging following ^90^Y RE; hence, the noise-level was clinically relevant.

### Statistical analysis

#### Phantom feature robustness study

Concordance correlation coefficient (CCC) metric assumed each observation was independent as has been commonly reported in repeatability/reproducibility studies [22, 32]. Thus, in the robustness study of our extracted radiomics features, CCCs were computed for the different scans, different iterations, and with/without Gaussian filtering. For each of the 45 radiomics features (without volume), the resulting CCCs were averaged, and features with larger than 0.85 [22, 33–36] average CCC-robust radiomics feature set were further investigated in the patient radiomics modeling.

#### Lesion overall response and progression modeling studies

1. Univariate analysis

Univariate association between the features (or absorbed dose) and OR classification was investigated using Spearman’s rank correlation. Univariate analysis for the features (or absorbed dose) and progression was investigated by Cox regression.

(2) Multivariate analysis—modified Bo-LASSO

**Fig. 1:**
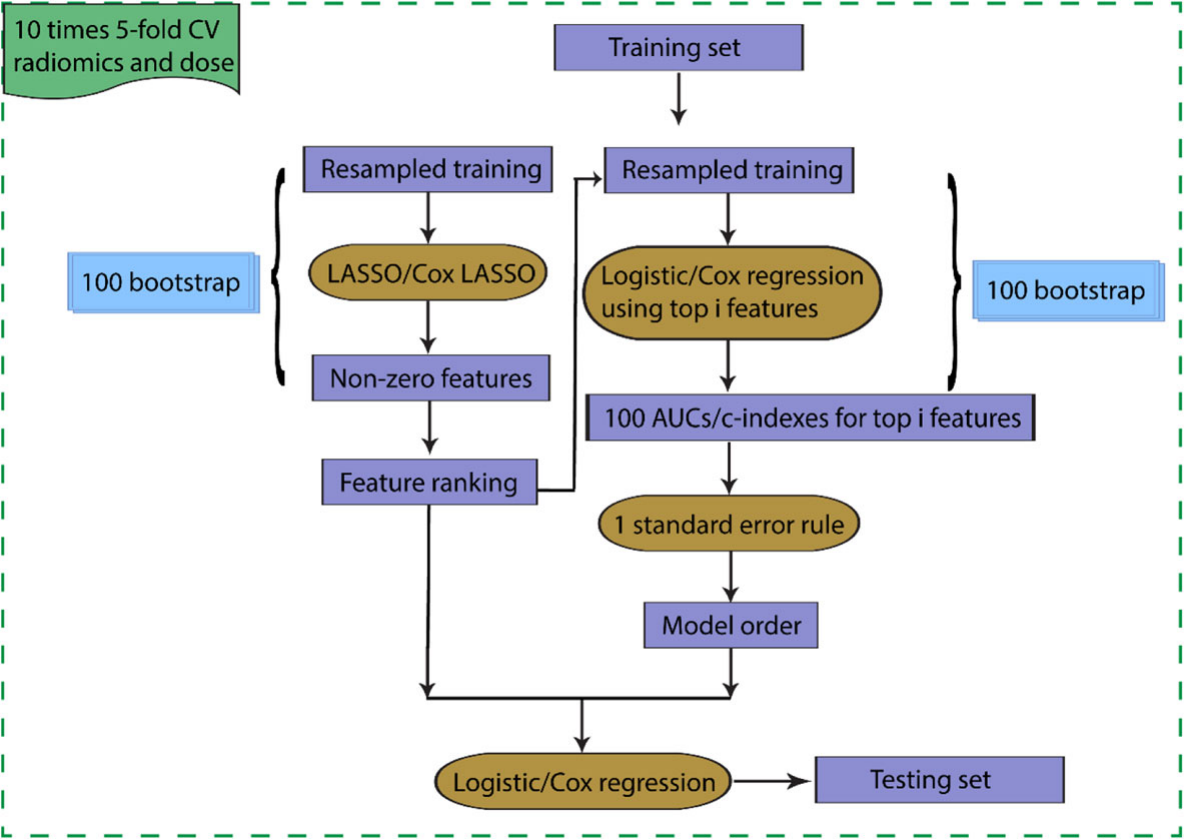
Summary of radiomics model construction and evaluation

In order to select robust features, build generalized models, and evaluate unbiased model performance, a nested cross-validation (CV) framework has been employed (details are shown in Fig. 1). In the outer loop, 10 times 5-fold cross validation was used to estimate the model performance. On the training set of each inner loop, *N* times bootstrap was performed. For each resampling training set, optimal lambda *λ* hyper-parameter for LASSO was tuned by another cross-validation process. Subsequently, features with non-zero coefficients were recorded. With *N* resampled training sets, *N* sets of features were recorded. The frequency of a certain feature being selected by LASSO was calculated, and thus, a ranking list of the features was obtained. Then, *M* times bootstrap logistic regression modeling was used to estimate the model order. Specifically, models using top *i* (*i* = 1, …, *n* = number of features) ranked features were developed, and mean AUC/c-index for each model order with confidence interval was obtained, and the model order corresponding to highest AUC/c-index within one standard error was selected [37]. After we obtained the model order and top selected features, final model in each outer loop was obtained by retraining on the training set and applied on the outer test set (here, *N* and *M* were both 100).

With the developed method, models were constructed using the 15 robust radiomics feature set, lesion volume, and mean absorbed dose (AD) (15 + 1 + 1 = 17). Since there are two subgroups in this patient cohort, the developed models were applied to both subgroups to assess if the tumor response correlated differently for primary HCC and metastatic lesions. The ROC curve (AUC) and c-index were used to evaluate the lesion OR and progression model performance, respectively. The confidence intervals were calculated by the bootstrap method [38]. The statistical analysis was performed using MATLAB R2019a and RStudio 1.1.463. The Bonferroni correction was applied to account for the family-wise error rate [39]. Overall, 17 features (dose + volume + 15 radiomics features) were tested; therefore, *p* values < 0.05/17 = 0.003 was considered significant. For the whole set of features (dose + volume + 45 radiomics features), *p* values < 0.05/47 = 0.001 were considered significant. Meanwhile, due to the existence of unbalance in the dataset, especially for progression analysis (events 14/103), adaptive synthetic sampling approach (ADASYN) was applied for the multivariate analysis to see if it can improve the performance [40].

## Results

### Phantom-based reproducibility and robustness of radiomics features

Supplemental Table 3 shows the mean CCC values from the liver phantom radiomics studies, assessed over the 5 repeat scans, OS-EM iterations 1/2, with/without Gaussian filtering, and across all conditions (scans and parameters). CCC for sphericity is always 1 because the shape feature does not depend on the PET scan. There are in total 15 features that have mean CCC > 0.85: 1 global feature sphericity; 1 GLCM feature correlation; 2 GLRLM features, gray-level nonuniformity (GLN) and run length nonuniformity (RLN); 7 GLSZM features, large zone emphasis (LZE), gray-level nonuniformity (GLN), zone size nonuniformity (ZSN), zone percentage (ZP), large zone low gray-level emphasis (LZLGE), large zone high gray-level emphasis (LZHGE), gray-level variance (GLV); 4 NGTDM features, coarseness, busyness, complexity, and strength. The average CCCs for repeatability (same conditions, different scans) and reproducibility (different iterations and filtering) have similar results as shown in Supplemental Table 3. Comparing with the mean CCC for both repeatability and reproducibility, there is 1 more robust feature for repeatability (dissimilarity), 6 more robust features (variance, contrast, dissimilarity, LGRE, SRLGE, GLV_GLRLM) and 2 less robust features (LZHGE, GLV_GLSZM) for different iterations, and 2 less robust features (ZSN, LZHGE) for with/without filtering.

### Lesion dosimetry and outcome data

A total of 105 lesions > 2 mL were segmented. The average lesion volume was 45 mL (median 10 ml, range 2–833). The average lesion absorbed dose was 336 Gy (median 265, range 1–1271). The response rate according to RECIST applied at the lesion level was 31% (32/105). The number of metastasis and primary HCC lesions is 70 and 35, respectively, with lesion-specific response rate being 26% (9/35) and 33% (23/70) for the 2 groups. There are 103 lesions that have progression data; two metastatic lesions were excluded due to lack of follow-up. The number of progression events for all the lesions was 14 (4 HCC, 10 metastatic). The mean time-to-event is 322 days (median 229 days, range 44–1174 days). The mean time-to-event was 342 days (median 309, range 50–1174) for metastatic lesions and 284 days (median 199 days, range 44–860 days) for HCC. Kaplan-Meier analysis showed that the time to progression for HCC and metastasis was not statistically significantly different (*P* = 0.49)

### Outcome models: radiomics, absorbed dose, and combined models

#### Univariate analysis

The univariate results for volume, radiomics features, and absorbed dose are shown in Supplemental Table 2 and Table 1 (with Supplemental Table 2 showing all the features and Table 1 showing only the 15 robust radiomics features). These are the Spearman correlation between specific features (or absorbed dose) and OR, and the univariate Cox regression results for progression. Volume has been shown to correlate with patient prognosis for different cancer types [41]. In our study, the Spearman coefficients of volume in terms of OR is − 0.215 (*p* value = 0.028). Among the 46 radiomics features (including volume), 10 features are significant (*p* value < 0.001) for OR: 2/9 GLCM features, 3/13 GLRLM features, 4/13 GLSZM features, and 1/5 NGTDM features. Among the 15 robust radiomics features, 8 features are significant for OR: LZE (*p* value = 0.0005), ZP (*p* value = 0.0004), LZLGE (*p* value = 0.001), LZHGE (*p* value = 0.002), GLV (*p* value = 0.0009), coarseness (*p* value = 0.003), busyness (*p* value = 0.001), and strength (*p* value = 0.003). Absorbed dose is a significant predictor of the OR (*p* value = 0.0003). In comparison, among the 46 radiomics features (including volume), no features are significant for progression. ZSN, a robust feature, is the most significant one (*p* value = 0.063) for progression. Absorbed dose is a marginally significant predictor for progression (*p* value = 0.005).

**Table 1:** Summary of statistical analysis for volume, the 15 robust radiomics features, and absorbed dose with Bonferroni correction

	Features	Spearman correlation with absorbed dose	***p*** value for dose correlation	Spearman correlation with OR	***p*** value for OR	C-index for progression	Hazard ratio for progression	***p*** value for progression
	**Volume**	− 0.262	0.007	− 0.215	0.028	0.565	0.282	0.417
**Global**	**Sphericity**	0.061	0.539	0.142	0.148	0.590	0.728	0.313
**GLCM**	**Correlation**	− 0.340	**3.882e**−**4**	− 0.216	0.027	0.438	1.019	0.950
**GLRLM**	**GLN**	− 0.362	**1.45e**−**4**	− 0.269	0.006	0.600	0.297	0.323
	**RLN**	− 0.252	0.010	− 0.236	0.015	0.639	0.213	0.201
**GLSZM**	**LZE**	− 0.482	**1.989e**−**7**	− 0.333	**0.0005**	0.562	0.415	0.629
	**GLN**	− 0.078	0.427	− 0.121	0.218	0.734	0.326	0.088
	**ZSN**	− 0.057	0.565	− 0.081	0.412	0.752	0.358	0.063
	**ZP**	0.483	**1.828e**−**7**	0.341	**0.0004**	0.491	0.804	0.502
	**LZLGE**	− 0.548	**1.485e**−**9**	− 0.317	**0.001**	0.460	0.872	0.760
	**LZHGE**	− 0.293	**0.002**	− 0.300	**0.002**	0.676	0.006	0.348
	**GLV**	0.467	**5.104e**−**7**	0.320	**0.0009**	0.549	0.491	0.136
**NGTDM**	**Coarseness**	0.379	**6.789e**−**5**	0.285	**0.003**	0.601	1.027	0.930
	**Busyness**	− 0.509	**2.862e**−**8**	− 0.307	**0.001**	0.482	0.522	0.585
	**Complexity**	0.324	**7.596e**−**4**	0.244	0.012	0.609	1.124	0.657
	**Strength**	0.245	0.012	0.284	**0.003**	0.669	1.110	0.321
**DOSE**	**Mean absorbed dose**	NA	NA	0.345	**0.0003**	0.819	0.121	0.005

**Fig. 2:**
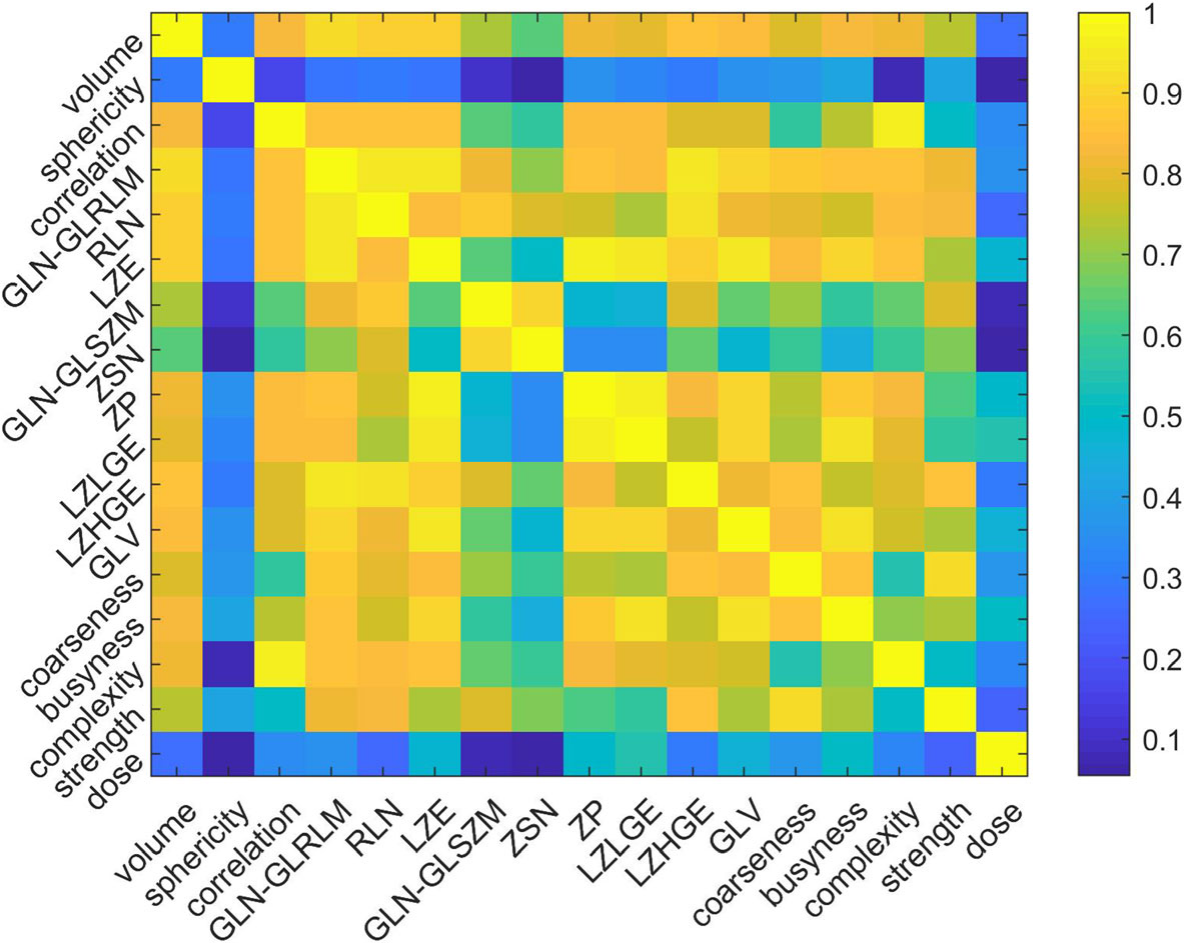
Spearman correlation heat map for radiomics features, volume and absorbed dose

Inter-feature correlation is shown in the correlation heat map of Fig. 2. GLN, RLN, LZE, and LZHGE are highly correlated with volume (Spearman coefficients > 0.85). In general, the radiomics features are highly correlated with each other (except sphericity). Though most of the radiomics features are still significantly correlated with dose (except sphericity, GLN, and ZSN), the correlation of radiomics features with dose is generally lower than radiomics features among them, as shown in Table 1.

#### Multivariate analysis

**Fig. 3:**
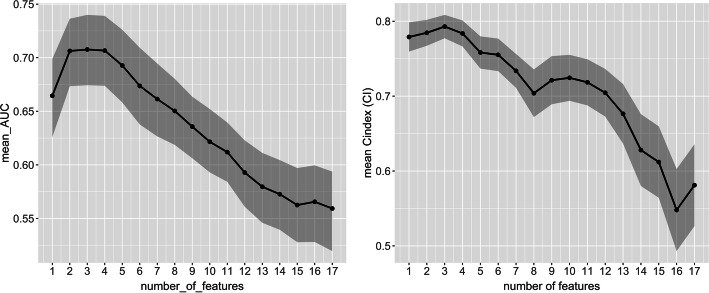
Radiomics_robust + dose model order determination for OR (left) and progression (right): average AUC/c-index vs. number of top features included

Given the limited sample size, we included both primary and metastasis cases in the modeling. For the subset of robust features, the model order is 2 for both OR and progression endpoints, with top 2 features for OR being absorbed dose and zone percentage (ZP), and for progression being absorbed dose and ZSN. Figure 3 shows the model order determination for the robust features and absorbed dose. The top 5 features are shown in Table 2 for OR and progression models. (Model order determination and the top 5 features using all the radiomics features and absorbed dose are presented in the supplemental materials Fig. 1 and Table 4.)

**Table 2:** Top 5 features for the combined models with robust radiomics features, volume, and absorbed dose

OR	Progression
Mean absorbed dose	Mean absorbed dose
ZP	ZSN
Sphericity	Strength
GLV	Complexity
Coarseness	Sphericity

**Fig. 4:**
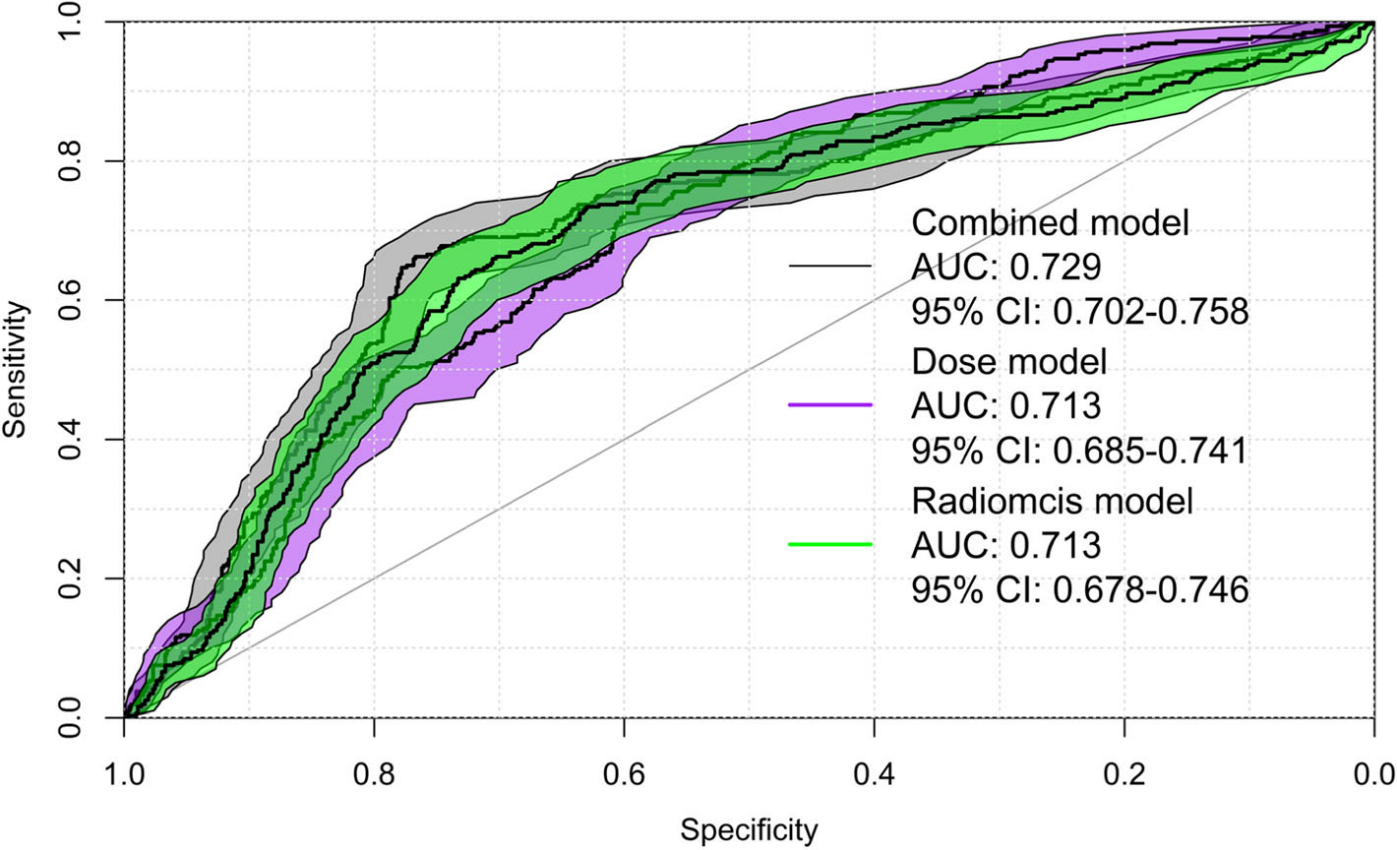
ROC curves for overall response (OR) at first follow-up with the combined model, radiomics alone model, and dose alone model

**Fig. 5:**
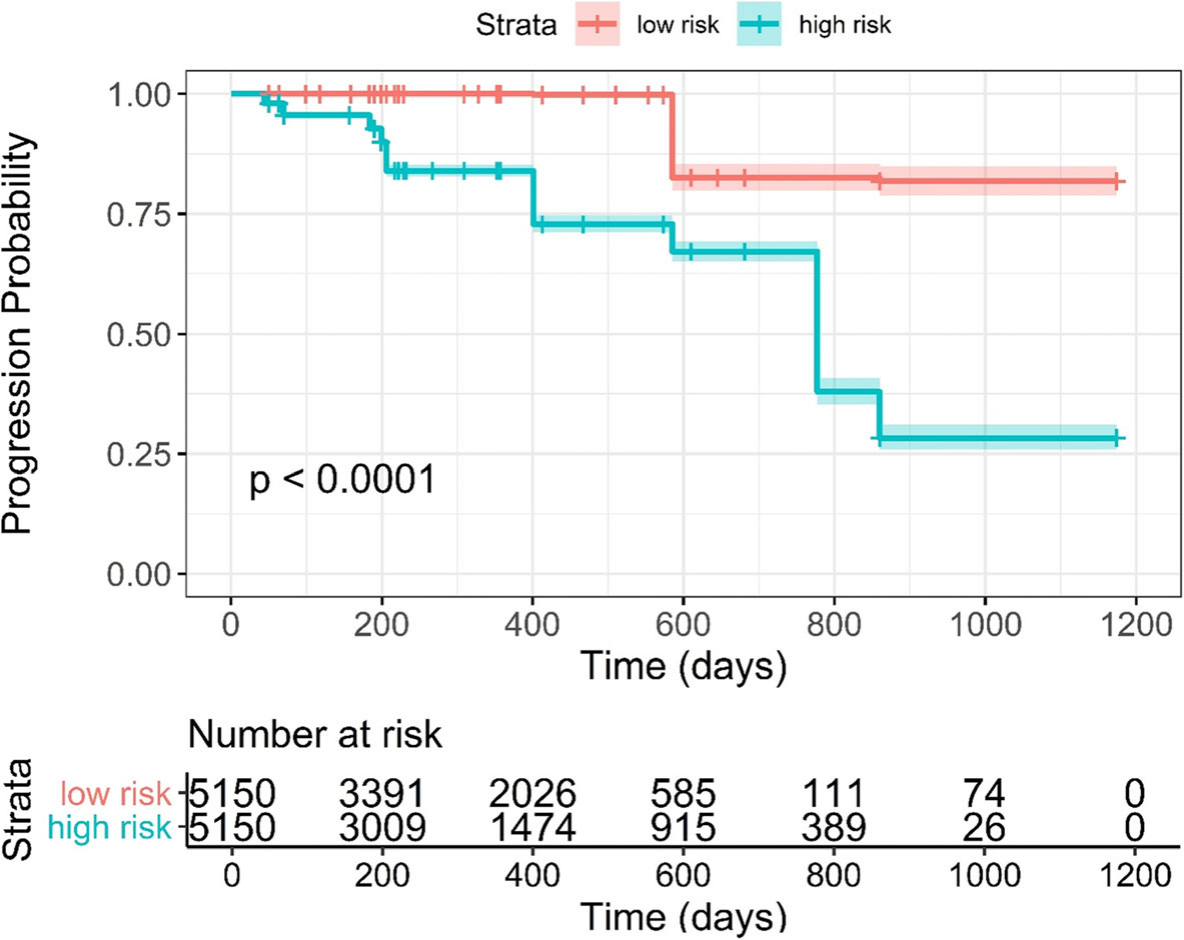
Kaplan-Meier plot of the combined model for progression (absorbed dose + ZSN). This is the result of 10 times 5-fold cross validation; thus, there are more test samples than the dataset. High and low risk lesions for progression were stratified by median value of the Cox model output, with high risk group lesions having shorter time to progression, vice versa

After the model order and top features were decided, nested cross-validation was applied to estimate the performance of the final model. The results for models with ZP only, ZSN only, absorbed dose only, and the combined models (radiomics robust + dose) are listed in Table 3. When considering the entire cohort, for the combined models, the average AUCs for OR (0.729 (95% CI 0.702–0.758)), and the average c-indexes for progression (0.803 (95% CI 0.790–0.815) are superior to the corresponding values for the absorbed dose only and ZP/ZSN only models. The results for the subgroups of primary and metastasis cases are shown in Table 3 as well. For the OR model in the subgroup of HCC, the radiomics only model shows the best performance with average AUC of 0.762 (95% CI 0.680–0.834), and in the subgroup of metastasis, the absorbed dose only model shows the best performance with average AUC of 0.696 (95% CI 0.654–0.737). For the progression analysis, in both subgroups, the combined model outperforms the individual models although the difference was not statistically significant. The ROC curve for OR using radiomics alone, dose alone, and combined models is shown in Fig. 4, and the Kaplan-Meier plot for progression for the combined models is shown in Fig. 5, respectively. Log-rank test was used for the comparison of high and low risk groups for progression. The cutoff was median value of the predicted Cox survival probability. The weights of OR model and progression models are shown below.

**Table 3:** Average AUC/c-index for individual and combined models with all the lesions, HCC lesions, and metastasis lesions

OR model	Average AUC (95 % confidence intervals)		
	All (105)	Primary HCC (35)	Metastasis (70)
**Radiomics (ZP)**	0.713 (0.685–0.741)	0.762 (0.680–0.834)	0.658 (0.623–0.693)
**Absorbed dose**	0.713(0.678–0.746)	0.717 (0.642–0.786)	0.696 (0.654–0.737)
**Combined (dose + ZP)**	0.729 (0.702–0.758)	0.734 (0.660–0.802)	0.692 (0.653–0.723)
**Progression model**	**Average c-index (95% confidence intervals)**		
	**All (103)**	**Primary HCC (35)**	**Metastasis (68)**
**Radiomics (ZSN)**	0.694 (0.676–0.710)	0.565 (0.528–0.598)	0.656 (0.629–0.680)
**Absorbed dose**	0.754 (0.742–0.766)	0.613 (0.585–0.635)	0.719 (0.700–0.737)
**Combined (dose + ZSN)**	0.803 (0.790–0.815)	0.638 (0.610–0.661)	0.762 (0.740–0.780)
**ADASYN progression model**	**Average c-index (95% confidence intervals)**		
	**All (103)**	**Primary HCC (35)**	**Metastasis (68)**
**Radiomics (ZSN)**	0.712 (0.698–0.726)	NA	0.595 (0.575–0.615)
**Absorbed dose**	0.771 (0.762–0.781)	NA	0.726 (0.713–0.739)
**Combined (dose + ZSN)**	0.794 (0.785–0.803)	NA	0.728 (0.716–0.740)

OR model (generalized linear regression model):

logit(y) ~ − 0.892 + 0.520 ZP + 0.488 dose

Distribution = binomial

Progression Cox model:

h(t) ~ h0(t) * exp(− 0.530 ZSN + − 1.707 dose)

**Fig. 6:**
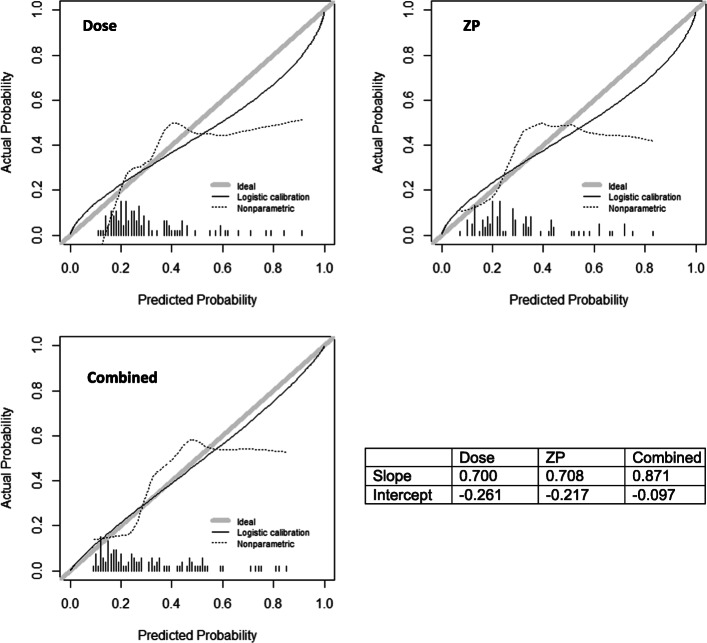
Calibration curves for dose, radiomics, and combined models for OR endpoint, with the slopes and intercepts available in the table

Artificially increasing the number of cases using ADASYN was evaluated for progression endpoint as well but found no substantial difference. Last part of Table 3 shows the results for ADASYN method. Figure 6 shows the calibration curves for OR models, with the calibration curves’ slope and intercept values available.

## Discussion

Uncovering robust radiomics features is an important task for building robust models for identifying responders and non-responders and prediction of cancer progression. Thus, radiomics features extracted from repeated PET scans, different number of OS-EM PET iterations, and with/without Gaussian post-filtering were evaluated for robustness using CCC. Despite the higher noise associated with ^90^Y PET compared with FDG PET, 15 radiomics features were identified as robust with CCC > 0.85. In general, the robust features for different scans (repeatability), OS-EM iterations 1/2, and with/without filtering largely overlap, which indicates that robust features tend to be consistent for different imaging settings. The results also showed that more features are robust to different iteration settings, and less features are robust to application of Gaussian filtering. In a study of intratumor FDG PET uptake heterogeneity quantification by Hatt et al., zone percentage (ZP) was found to be robust with respect to the delineation method used and the partial volume effects. This feature also demonstrated high differentiation power for prediction of response in esophageal carcinoma [42]. In a study by Doumou et al., ZP presented substantial agreement across different segmentation and different levels of smoothing [43]. A study by Ashrafinia et al. showed that ZSN extracted from ^99m^Tc-Sestamibi Myocardial-Perfusion SPECT (MPS) images showed high reproducibility [44]. Another recent study by Li et al. on FDG PET radiomics analysis showed that ZSN is a stable feature [45]. The phantom repeatability and reproducibility study provides robust features for further radiomics modeling that has the potential to generalize to PET images reconstructed at other institutions where different reconstruction settings might have been applied. While this phantom study focused on reconstruction settings, there are other sources of variability as mentioned that we did not evaluate here, such as segmentation, interpolation, and preprocessing, which are investigated in other literatures [22, 23, 33, 46] and reviewed in [47, 48].

The aim of this work is to find radiomics signature that can facilitate dose metrics in the prediction of tumor response. The final model order is small being 2 (dose + ZP and dose + ZSN), which is reasonable considering the high correlation between most radiomics features (Fig. 2). The correlation between ZP and absorbed dose is 0.483 (*p* value = 1.828e−7) and ZSN and absorbed dose is − 0.057 (*p* value = 0.565) (Table 1), which indicates that ZSN could provide more complementary information to the combined model than ZP. This is consistent with the substantial higher c-index for the combined absorbed dose and ZSN model (0.803) compared with ZSN only (0.694) and absorbed dose only (0.754) models for progression, but only slightly higher AUC for the combined absorbed dose and ZP model (0.729) compared with the ZP only (0.713) and absorbed dose only (0.713) models for OR (Table 3). In Fig. 4, the ROC curves for radiomics alone, dose alone, and combined models did present some overlap. However, it still showed consistent trends in the data that the combined model can perform better than individual models. Furthermore, we plotted the calibration curves of the OR models as shown in Fig. 6. For calibration curve, a slope of 1 and intercept of 0 is the ideal situation. In terms of the slope, combined model is 0.871, which is closer to 1 comparing with dose (0.700) and ZP (0.708) alone. For the calibration intercept, combined model (− 0.097) is closer to 0 than dose (− 0.261) and ZP (− 0.217). This calibration result showed that although the combined model is not significantly better than individual models for the discrimination (AUC) power, it is trending in the right direction and with larger sample size may attain the desired level of significance. Access to larger Y-90 PET imaging datasets is required to independently validate these findings and to reach desired statistical significance for the improvement of the performance of the combined model over the individual models, which was not established in the current study. The absence of external validation is a limitation of the current study. Further studies, such as obtaining radiomics features from FDG-PET, CT, or MRI, could potentially add more complementary information and further improve the performance [49].

Due to imbalance of progression endpoint (14 events out of 103), we did ADASYN, a variant of SMOTE. The difference for ADASYN with SMOTE is that it does not simply interpolate the minority samples to generate synthetic cases, it adaptively generates minority data samples according to their distributions using K nearest neighbor. The primary subgroup has 4 events, and it is inaccurate to do ADASYN (which is based on KNN methods); thus, the ADASYN was only applied to all data and the mets subgroup. The difference is subtle comparing the results based on original dataset and the ADASYN balanced dataset. A larger dataset will potentially enhance the models or make complex algorithms work better.

**Fig. 7:**
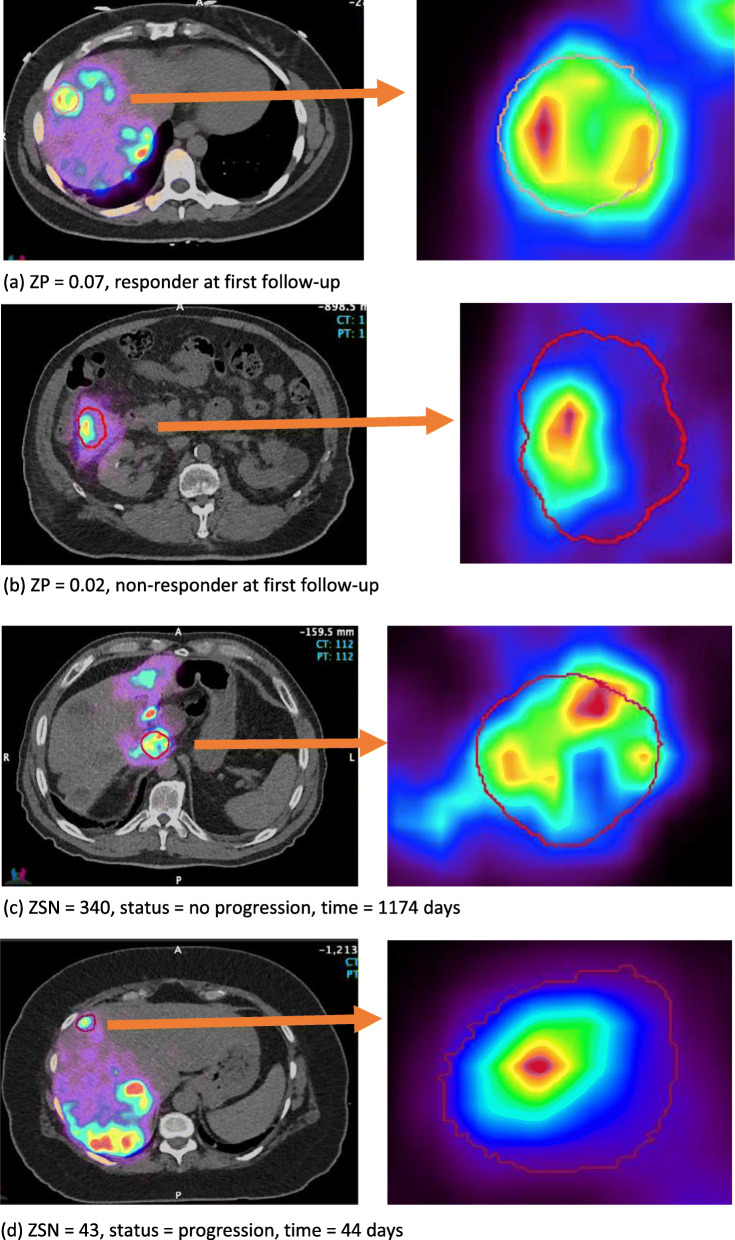
Example ^90^Y PET/CT images with CT-defined lesion contours (left: PET/CT axial slice showing the anatomical position within liver, right: magnified lesion on PET). **a** Lesion with large ZP value corresponding to responder. **b** Lesion with small ZP value corresponding to non-responder. **c** Lesion with large ZSN value corresponding to no progression at 1174 day. **d** Lesion with small ZSN value corresponding to progression in 44 days

ZP is a feature from GLSZM matrix, quantifying the coarseness of the texture by the ratio of number of zones and number of voxels. The higher the value is, the finer the texture is, and according to our results, the higher the probability the tumor will respond. Figure 7a and b show example lesions with large/small ZP that were classified as responder/non-responder; c and d show lesions with large/small ZSN that did not progress for a long follow-up time (1174 days) and progressed in a short time (44 days). Smaller ZP values correspond to coarser appearance and worse response. In another study by Ha et al., ZP was one of the features used to characterize locally advanced breast cancer [50]. The trend is consistent with what we found in our study that larger ZP is associated with better response. ZSN measures the variability of size zone volumes in the ROIs; the higher the value, the larger the variance of the size zone volumes. The hazard ratio for ZSN is smaller than 1, which means the higher the ZSN, the better the lesion prognosis.

The modified LASSO method we developed was inspired by R. Bach’s work on Bolasso, which showed that the Lasso selects all the variables that should enter the model with probability tending to one exponentially fast [51]. So, if we run the Lasso for multiple bootstrapped replications of a given sample, then, intersecting the supports of the Lasso (i.e., non-zero coefficients) leads to consistent model selection. However, the direct application failed since the intersection of the supports lead to null for some datasets. Bunea et al. came up with similar variants of bootstrap enhanced LASSO (BE-LASSO) [52]. The percentage of times each predictor was selected (variable inclusion probability) was recorded, and user-defined threshold (50%) was used to determine the variables. Abram et al. built upon Bunea’s method of Be-LASSO [53]. Instead of user-defined probability for feature selection, they used the quantiles of the bootstrap distribution of the coefficients of variables to determine the significance of that variable. In our study, we developed a new way to select features, still based on the bootstrap LASSO. Instead of using predefined probability or the distribution quantile, we obtained a ranking of the features based on the frequency of being selected in the bootstrap; then, we performed cross validation to calculate the AUC/c-index vs. number of top features included in the model. In this way, we obtained the most parsimonious model, which is desired when small sample size is unavoidable.

The features that we included in this study (1 shape, 5 global and 10 GLCM, 13 GLRLM, 13 GLSZM, and 5 NGTDM features) are not the whole spectrum of the existing radiomics features. However, in this study, our focus is textural patterns (second order radiomics features), and we did cover all the 5 NGTDM features, most of the GLRLM and GLSZM features. For GLCM features, although there are more features, we covered the main ones [6, 54–57]; others are variations of the main type. The first order features that we considered (variance, coefficient of variation, skewness, and kurtosis) are calculated by subtracting the mean of the intensity values, which removes the influence of the absolute intensity values. Examples of first order features that we did not use are energy, entropy, and minimum, which depend on the absolute intensity values. In addition to sphericity and volume that we investigated, there are some other 3D shape features, such as surface area, surface to volume ratio, and compactness, that we did not investigate. However, these are mostly functions of volume and surface area. In summary, we limited the radiomics features to cover the main ones for each category, and the first order non-stable features were avoided due to lack of robustness. Although using more features may be desirable, in this study, we limited the feature size to be as non-redundant and robust as possible to relieve the burden of feature selection afterwards. In addition, many of the extensive list of features are parametrization of these basic ones, and with the limited sample size, they are unlikely to provide significant advantage at this stage.

In summary, absorbed dose is a strong predictor for tumor control, both in terms of OR at first follow-up and time to progression, which is consistent with recent reports [14–16]. The radiomics feature signals the complimentary value of texture to improve the absorbed dose only model prediction. It is interesting to explore the underlying biological mechanism of the reason for higher ZP and ZSN leading to better prognosis, which should be investigated on larger dataset in the future. The two-feature model can be interpreted as given the dose being fixed, the change in ZP/ZSN will help to predict tumor control (OR/progression). Using this information, additional attentions would be given to the lesions that possess lower ZP/ZSN value, which have a higher risk of failure (in terms of OR/progression), which is potentially informative for clinical decisions. Immediate prediction of response, based on radiomics features and dose metrics both of which can be derived from ^90^Y PET/CT performed immediately after RE, has clinical utility. Instead of waiting for the first follow-up morphologic imaging that typically occurs at > 2 months, the potential to predict non-responding lesions immediately after therapy would facilitate adaptive therapy to selected lesions where ^90^Y RE is followed by further treatment such as stereotactic body radiation therapy or microwave ablation. Limitation of our study include the heterogeneous patient cohort, the small sample size, and not achieving statistical significance showing that OR response models that combine absorbed dose and radiomics are superior to models based on absorbed dose or radiomics alone. Patient ^90^Y imaging data is scarce because post-therapy imaging is not routinely performed after RE, but studies reporting ^90^Y SPECT/CT and PET/CT imaging is rising and is expected to become more readily available, enabling studies with larger cohorts in the future to address limitations of the current study.

## Conclusion

In this study, radiomics only, absorbed dose only, and combined models showed predictive ability for tumor OR and progression in ^90^Y radioembolization patients. The final tumor OR model consisting of the robust radiomics feature ZP and mean absorbed dose achieved a nested CV AUC 0.729 while the final progression model consisting of the robust radiomics feature ZSN and mean absorbed dose achieved a c-index of 0.803. Further validation on larger external cohorts will be necessary to statistically establish the superiority of the combined model, which was not achieved in the current study. Nonetheless, this study showed the potential of combining ^90^Y PET-derived radiomics and absorbed dose for improved model building to predict tumor OR and progression in ^90^Y radioembolization treatment.

## Supplementary Information

**Additional file 1.** Supplemental Table 1. Patient/lesion characteristics of the cohort and sub-cohort (HCC and metastasis). Supplemental Table 2. Spearman correlation coefficients between lesion-level overall response and all radiomics features/absorbed dose with corresponding p-values (with Bonferroni correction). Univariate Cox regression with c-index, hazard ratio and corresponding p-values for progression are also indicated. Supplemental Table 3. Mean CCC for 5 repeat scans of the liver phantom, OS-EM iterations 1/2, with/without Gaussian filtering and across all conditions. Supplemental Fig. 1 Radiomics_all+dose model order determination for OR (left) and PFS (right). Average AUC/c-index vs. number of top features included. When using all the radiomics features, the average model order calculated using nested cross validation is 2 for OR classification and 3 for PFS, with top 2 features being variance and absorbed dose and top 3 features being variance, absorbed dose and LRHGE, respectively. The nested CV AUCs for radiomics_all+dose is 0.672 (0.620-0.716) for OR and 0.791 (95%CI: 0.740-0.825) for progression. The top 5 features are shown in supplemental table 4 for OR and progression models. Supplemental Table 4. Top 5 features for the combined models with all radiomics features, volume and absorbed dose.

## Data Availability

Anonymized ^90^Y PET/CT DICOM data including segmented lesions for select patients are available at the University of Michigan Library Deep Blue repository: 10.7302/v07v-z854and 10.7302/pf4m-vn04 Radiomics extraction code implemented in this work is shared under the GNU General Public License at: https://github.com/mvallieres/radiomics.
