# Supplementary material for: Tumor response prediction in 90Y radioembolization with PET-based radiomics features and absorbed dose metrics
Source: EJNMMI Phys. 2020 Dec 9;7:74. doi: 10.1186/s40658-020-00340-9 (PMC7726084; doi:10.1186/s40658-020-00340-9)
Supplement: Supplementary file 1 — Additional file 1. Supplemental Table 1. Patient/lesion characteristics of the cohort and sub-cohort (HCC and metastasis). Supplemental Table 2. Spearman correlation coefficients between lesion-level overall response and all radiomics features/absorbed dose with corresponding p-values (with Bonferroni correction). Univariate Cox regression with c-index, hazard ratio and corresponding p-values for progression are also indicated. Supplemental Table 3. Mean CCC for 5 repeat scans of the liver phantom, OS-EM iterations 1/2, with/without Gaussian filtering and across all conditions. Supplemental Fig. 1 Radiomics_all+dose model order determination for OR (left) and PFS (right). Average AUC/c-index vs. number of top features included. When using all the radiomics features, the average model order calculated using nested cross validation is 2 for OR classification and 3 for PFS, with top 2 features being variance and absorbed dose and top 3 features being variance, absorbed dose and LRHGE, respectively. The nested CV AUCs for radiomics_all+dose is 0.672 (0.620-0.716) for OR and 0.791 (95%CI: 0.740-0.825) for progression. The top 5 features are shown in supplemental table 4 for OR and progression models. Supplemental Table 4. Top 5 features for the combined models with all radiomics features, volume and absorbed dose. [file 40658_2020_340_MOESM1_ESM.docx]

Supplemental Table 1. Patient/lesion characteristics of the cohort and sub-cohort (HCC and metastasis).

|  | **Cohort** |
| --- | --- |
| Disease |  |
| Primary HCC | 13 (43%) |
| Liver Metastasis | 17 (57%) |
| Total Patients | 30 |
| Total Therapies | 36 |
|  |  |
| Number of lesions |  |
| HCC | 35 (33%) |
| Liver Metastasis | 70 (67%) |
| Total Lesions | 105 |
|  |  |
| Cirrhotic livers | 11 (37%) |
|  |  |
| Lesion volume (mL) | Median [range] |
| Primary HCC | 11.5 [2.1-204] |
| Liver Metastasis | 9.3 [2.2-833] |
|  |  |
| Number of lesions per patient | 3 [1-5] |

Supplemental Table 2. Spearman correlation coefficients between lesion-level overall response and all radiomics features/absorbed dose with corresponding p-values (with Bonferroni correction). Univariate Cox regression with c-index, hazard ratio and corresponding p-values for progression are also indicated.

|  | **Radiomics and other metrics** | **Spearman correlation for OR** | **P value for OR** | **C-index for progression** | **Hazard Ratio for progression** | **P value for progression** |
| --- | --- | --- | --- | --- | --- | --- |
|  | **Volume** | -0.215 | 0.028 | 0.565 | 0.282 | 0.417 |
| **Global** | **Sphericity** | 0.142 | 0.148 | 0.590 | 0.728 | 0.313 |
|  | **Variance** | -0.128 | 0.193 | 0.789 | 0.193 | 0.002 |
|  | **Coefficient of variation** | -0.241 | 0.013 | 0.657 | 1.129 | 0.298 |
|  | **Skewness** | -0.161 | 0.100 | 0.741 | 1.298 | 0.116 |
|  | **Kurtosis** | 0.133 | 0.177 | 0.657 | 1.147 | 0.226 |
| **GLCM** | **Energy** | -0.137 | 0.164 | 0.537 | 1.103 | 0.333 |
|  | **Contrast** | 0.247 | 0.011 | 0.484 | 0.885 | 0.711 |
|  | **Entropy** | 0.212 | 0.030 | 0.484 | 0.837 | 0.158 |
|  | **Homogeneity** | -0.348 | **0.0003** | 0.489 | 1.275 | 0.233 |
|  | **IDM** | -0.351 | **0.0002** | 0.488 | 1.280 | 0.240 |
|  | **Correlation** | -0.216 | 0.027 | 0.438 | 1.019 | 0.950 |
|  | **SumMean** | 0.206 | 0.035 | 0.747 | 0.651 | 0.023 |
|  | **Variance_GLCM** | -0.069 | 0.487 | 0.704 | 0.588 | 0.037 |
|  | **Dissimilarity** | 0.277 | 0.004 | 0.484 | 0.858 | 0.609 |
| **GLRLM** | **SRE** | 0.368 | **0.0001** | 0.532 | 0.744 | 0.169 |
|  | **LRE** | -0.374 | **8.321e-05** | 0.482 | 1.109 | 0.361 |
|  | **GLN** | -0.269 | 0.006 | 0.600 | 0.297 | 0.323 |
|  | **RLN** | -0.236 | 0.015 | 0.639 | 0.213 | 0.201 |
|  | **RP** | 0.366 | **0.0001** | 0.507 | 0.791 | 0.197 |
|  | **LGRE** | -0.121 | 0.218 | 0.702 | 1.140 | 0.213 |
|  | **HGRE** | 0.183 | 0.061 | 0.775 | 0.538 | 0.011 |
|  | **SRLGE** | -0.055 | 0.578 | 0.737 | 1.299 | 0.073 |
|  | **SRHGE** | 0.213 | 0.029 | 0.754 | 0.532 | 0.015 |
|  | **LRLGE** | -0.226 | 0.021 | 0.644 | 1.077 | 0.481 |
|  | **LRHGE** | -0.031 | 0.753 | 0.820 | 0.674 | 0.136 |
|  | **GLV** | 0.269 | 0.006 | 0.633 | 0.507 | 0.097 |
|  | **RLV** | 0.295 | 0.002 | 0.563 | 0.577 | 0.169 |
| **GLSZM** | **SZE** | 0.072 | 0.465 | 0.745 | 0.558 | 0.006 |
|  | **LZE** | -0.333 | **0.0005** | 0.562 | 0.415 | 0.629 |
|  | **GLN** | -0.121 | 0.218 | 0.734 | 0.326 | 0.088 |
|  | **ZSN** | -0.081 | 0.412 | 0.752 | 0.358 | 0.063 |
|  | **ZP** | 0.341 | **0.0004** | 0.491 | 0.804 | 0.502 |
|  | **LGZE** | -0.010 | 0.920 | 0.663 | 1.438 | 0.156 |
|  | **HGZE** | -0.034 | 0.732 | 0.629 | 0.919 | 0.733 |
|  | **SZLGE** | 0.039 | 0.691 | 0.613 | 1.194 | 0.453 |
|  | **SZHGE** | -0.004 | 0.970 | 0.764 | 0.586 | 0.058 |
|  | **LZLGE** | -0.317 | **0.001** | 0.460 | 0.872 | 0.760 |
|  | **LZHGE** | -0.300 | 0.002 | 0.676 | 0.006 | 0.348 |
|  | **GLV** | 0.320 | **0.0009** | 0.549 | 0.491 | 0.136 |
|  | **ZSV** | -0.233 | 0.017 | 0.601 | 1.383 | 0.007 |
| **NGTDM** | **Coarseness** | 0.285 | 0.003 | 0.601 | 1.027 | 0.930 |
|  | **Contrast** | 0.228 | 0.020 | 0.518 | 0.725 | 0.448 |
|  | **Busyness** | -0.307 | **0.001** | 0.482 | 0.522 | 0.585 |
|  | **Complexity** | 0.244 | 0.012 | 0.609 | 1.124 | 0.657 |
|  | **Strength** | 0.284 | 0.003 | 0.669 | 1.110 | 0.321 |
| **Dose** | **Mean absorbed dose** | 0.345 | **0.0003** | 0.819 | 0.121 | 0.005 |

Supplemental Table 3. Mean CCC for 5 repeat scans of the liver phantom, OS-EM iterations 1/2, with/without Gaussian filtering and across all conditions.

|  | **Radiomics and other metrics** | **Mean CCC for 5 scans** | **Mean CCC for iterations 1/2** | **Mean CCC for with/without Gaussian filtering** | **Mean CCC across all conditions** |
| --- | --- | --- | --- | --- | --- |
|  | **Volume** | NA | NA | NA | NA |
| **Global** | **Sphericity** | **1.000** | **1.000** | **1.000** | **1.000** |
|  | **Variance** | 0.465 | **0.957** | 0.439 | 0.621 |
|  | **Coefficient of variation** | 0.567 | 0.689 | 0.499 | 0.585 |
|  | **Skewness** | 0.384 | 0.724 | 0.855 | 0.654 |
|  | **Kurtosis** | 0.228 | 0.415 | 0.501 | 0.381 |
| **GLCM** | **Energy** | 0.183 | 0.747 | 0.144 | 0.358 |
|  | **Contrast** | 0.825 | **0.931** | 0.695 | 0.817 |
|  | **Entropy** | 0.239 | 0.712 | 0.336 | 0.429 |
|  | **Homogeneity** | 0.682 | 0.835 | 0.732 | 0.750 |
|  | **IDM** | 0.627 | 0.821 | 0.717 | 0.722 |
|  | **Correlation** | **0.897** | **0.967** | **0.922** | **0.929** |
|  | **SumMean** | 0.158 | 0.686 | 0.134 | 0.326 |
|  | **Variance_GLCM** | 0.689 | 0.640 | 0.485 | 0.604 |
|  | **Dissimilarity** | **0.869** | **0.912** | 0.806 | 0.845 |
| **GLRLM** | **SRE** | 0.517 | 0.787 | 0.642 | 0.648 |
|  | **LRE** | 0.629 | 0.788 | 0.537 | 0.651 |
|  | **GLN** | **0.998** | **0.998** | **0.996** | **0.997** |
|  | **RLN** | **0.997** | **0.997** | **0.991** | **0.995** |
|  | **RP** | 0.611 | 0.814 | 0.654 | 0.693 |
|  | **LGRE** | 0.056 | **0.867** | 0.186 | 0.370 |
|  | **HGRE** | 0.145 | 0.706 | 0.174 | 0.342 |
|  | **SRLGE** | 0.051 | **0.885** | 0.152 | 0.363 |
|  | **SRHGE** | 0.279 | 0.811 | 0.269 | 0.453 |
|  | **LRLGE** | 0.077 | 0.814 | 0.302 | 0.398 |
|  | **LRHGE** | 0.280 | 0.333 | 0.184 | 0.266 |
|  | **GLV** | 0.674 | **0.934** | 0.679 | 0.762 |
|  | **RLV** | 0.633 | 0.845 | 0.500 | 0.659 |
| **GLSZM** | **SZE** | 0.046 | 0.370 | 0.441 | 0.286 |
|  | **LZE** | **0.948** | **0.910** | **0.879** | **0.912** |
|  | **GLN** | **0.953** | **0.985** | **0.988** | **0.975** |
|  | **ZSN** | **0.928** | **0.895** | 0.839 | **0.887** |
|  | **ZP** | **0.880** | **0.919** | **0.927** | **0.909** |
|  | **LGZE** | -0.023 | 0.703 | -0.174 | 0.169 |
|  | **HGZE** | 0.384 | 0.606 | 0.556 | 0.515 |
|  | **SZLGE** | 0.091 | 0.832 | -0.161 | 0.254 |
|  | **SZHGE** | 0.042 | 0.342 | -0.067 | 0.106 |
|  | **LZLGE** | **0.885** | **0.978** | **0.949** | **0.937** |
|  | **LZHGE** | **0.877** | 0.834 | 0.719 | **0.850** |
|  | **GLV** | **0.880** | 0.782 | **0.870** | **0.862** |
|  | **ZSV** | 0.541 | 0.724 | 0..659 | 0.641 |
| **NGTDM** | **Coarseness** | **0.920** | **0.982** | **0.922** | **0.941** |
|  | **Contrast** | 0.733 | 0.841 | 0.424 | 0.666 |
|  | **Busyness** | **0.940** | **0.958** | **0.909** | **0.936** |
|  | **Complexity** | **0.893** | **0.988** | **0.924** | **0.935** |
|  | **Strength** | **0.958** | **0.983** | **0.921** | **0.954** |
| **Dose** | **Mean absorbed dose** | NA | NA | NA | NA |


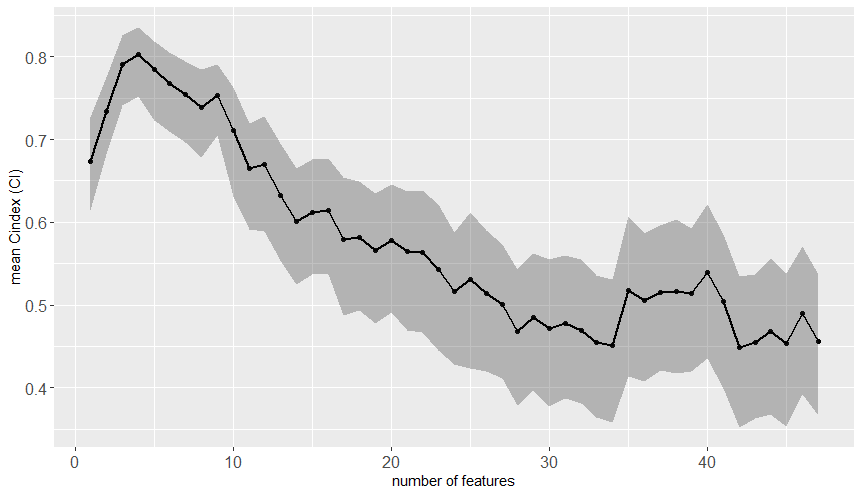

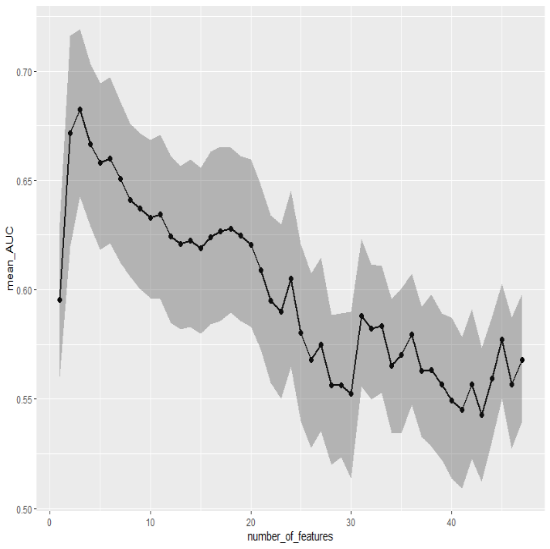


Supplemental Fig. 1 Radiomics_all+dose model order determination for OR (left) and PFS (right). Average AUC/c-index vs. number of top features included. When using all the radiomics features, the average model order calculated using nested cross validation is 2 for OR classification and 3 for PFS, with top 2 features being variance and absorbed dose and top 3 features being variance, absorbed dose and LRHGE, respectively. The nested CV AUCs for radiomics_all+dose is 0.672 (0.620-0.716) for OR and 0.791 (95%CI: 0.740-0.825) for progression. The top 5 features are shown in supplemental table 4 for OR and progression models.

Supplemental Table 4. Top 5 features for the combined models with all radiomics features, volume and absorbed dose

| **OR** | **Progression** |
| --- | --- |
| Variance | Variance |
| Mean absorbed dose | Mean absorbed dose |
| Sphericity | LRHGE |
| SZE | SZHGE |
| LRHGE | Kurtosis |
